# Correction to *Lancet Diabetes Endocrinol* 2018; 6: 82–83

**DOI:** 10.1016/S2213-8587(18)30135-9

**Published:** 2018-06

*Park Y, Colditz GA. Diabetes and adiposity: a heavy load for cancer.* Lancet Diabetes Endocrinol *2018; **6:** 82–83*—In this Comment, the third, fourth, fifth, and sixth sentences of the second paragraph should read “5·7% of all cancer in 2012 was attributable to high BMI and diabetes in 2002. When the estimation was restricted to 12 obesity-related cancers and six diabetes-related cancers, 15·3% of cancer cases in men and 13·5% of cases in women were due to the combined effect of high BMI and diabetes. However, there was substantial variation in a proportion of cancer due to high BMI and diabetes by cancer sites—for example, 23·3% of liver cancer, 18·0% of pancreatic cancer, and 9·5% of colorectal cancer cases in men were attributable to the combined effect of high BMI and diabetes. Pearson-Stuttard and colleagues suggest that 25·8% of diabetes-related cancers and 31·9% of cancers related to high BMI in 2012 were due to increases in the prevalence of these risk factors from 1980 to 2002.” The reference for the study to which this Comment relates has also been updated. These corrections have been made to the online version as of May 23, 2018.

